# Non-Invasive Acoustic Monitoring of Gas Turbine Units by Fiber Optic Sensors

**DOI:** 10.3390/s22134781

**Published:** 2022-06-24

**Authors:** Konstantin V. Stepanov, Andrey A. Zhirnov, Stanislav G. Sazonkin, Alexey B. Pnev, Alexander N. Bobrov, Dmitriy A. Yagodnikov

**Affiliations:** 1 Bauman Moscow State Technical University, 2-nd Baumanskaya 5-1, 105005 Moscow, Russia; a.zh@bmstu.ru (A.A.Z.); sazstas@bmstu.ru (S.G.S.); pniov@bmstu.ru (A.B.P.); abbvv@bmstu.ru (A.N.B.); daj@bmstu.ru (D.A.Y.); 2 Kotelnikov Institute of Radioengineering and Electronics of RAS, Mokhovaya 11-7, 125009 Moscow, Russia

**Keywords:** Mach–Zehnder interferometer, fiber optic sensor, gas turbine unit, acoustic monitoring, non-invasive monitoring

## Abstract

In this article, we study the possibility of gas turbine unit (GTU) monitoring using interferometric fiber optic sensors. We used the Mach–Zehnder interferometer (MZI) scheme, which can be easily implemented and simply installed on the turbine, and also allows us to solve the problem of phase unwrapping conveniently. In this research, the following main steps were carried out: an experimental scheme based on the MZI was assembled, and its sensitive arm was fixed on the GTU under study; data on various operation modes of the GTU was collected; the data were subjected to frequency FFT analysis, based on which the main rotational speeds of the turbine were identified. With FFT analysis, we also demonstrated multiples harmonics, which appear in the case of GTU after operating time, caused by the number of blades. The possibility of GTU monitoring and analysis using a non-invasive compact fiber-optic sensor is demonstrated: spectral analysis is used to detect the rotor speed, as well as the presence or absence of high-order multiple frequencies indicating blade and bearing defects, which are determined by the number of GTU’s blades and rolling bearing used as turbines rotor supports.

## 1. Introduction

Gas turbines (GTs), due to their high specific characteristics, are in-demand engines for various gas turbine units (GTUs). They are widely used in gas pumping units for the gas industry and in systems used in shipbuilding, aviation, and space rocket industries, where low weight and dimensions of units are essential. For example, GTs are used as a drive unit for water jet propulsion, screw-propellers, fans, blade compressors, pumps for hydraulic systems, and fuel supply systems in liquid rocket engines, spacecraft, power ships, and jet aircraft propulsion systems. There are also known works on the use of GT as the main drive unit and supercharging systems for engines in vehicular transport [1]. The significant contribution of GTUs to the overall efficiency of the system, its reliability, and its durability make the problems of diagnosing their technical condition [2] by various means relevant.

Currently, there are several approaches to the implementation of systems for technical diagnostic of GTUs. The most widely used method is based on the use of staff instrumentation, which causes additional costs for the diagnostic system’s design and manufacture and increases the mass of the GTU. Also, the operability ensuring requirements over the entire range of operating loads often leads to their rather low accuracy, frequently allowing only the detection of the malfunction occurrence [2].

Another way is the method of indirect parametric diagnostics, which can be characterized as object monitoring by evaluating a selected parameter, which is directly related to the functional purpose of the object and directly characterizes its technical condition. This method can be applied to those not equipped with standard diagnostic systems GTUs or to improve the accuracy of staff systems, for example, for diagnosing overpressure, pressure drop, temperature, rotor speed, thrust, and others [3,4,5]. In article [6], the authors describe the use of a diagnostic model for the state of the combustion chamber, turbine, and centrifugal compressor. An analysis of the diagnostic of effective power and efficiency of the GTU was proposed in [7,8,9]. In recent years, the usage of computer neural networks has been growing for parametric analysis of the technical systems state [10,11,12,13,14,15].

It is worth highlighting the vibration diagnostic method. During the operation of the GTU, cyclic processes occur, such as the rotation of the turbine rotor and periodic loads on the unit’s elements, which lead to vibrations. Various defects, such as uneven deterioration, destruction of blades or bearings, and imbalance of the rotor, change the cyclic processes, which leads to an inevitable change in the vibroacoustic response [16]. Analysis of vibration signals and their spectra makes it possible to detect the main mechanical defects: imbalance of rotating masses; rotor misalignment of GTU; bearing failure; touching the stator by the rotor. When analyzing the causes of GTU’s failures and defects, it is necessary to measure not only absolute but also relative components of vibroacoustic signals [17]. The results of technical diagnostics of the working blade’s destruction of a stationary GTU are presented in [18]. Vibration diagnostics were used for the detection of rumble and screech events on engines in a test cell [19], analysis of vibration parameters of ship GT engines [20], blade monitoring [21], vibration limits for non-rotating parts [22], aircraft GT engine [23,24], rotary machines monitoring [25]. Recently, vibration sensors based on fiber optic technologies have gained increasing interest.

Fiber optic sensors and fiber optic systems (FOS) based on them, due to the increase in the range and improvement of the characteristics of components, such as the development and commercial implementation of narrow-band laser sources, low-noise receivers, and high-speed ADCs, are not inferior, and in some applications, they surpass similar classical primary measuring transducers (PMT). FOSs have a number of advantages over PMTs, for example, fiber optic sensors do not require a power supply for fiber transducers, which means its complete explosion and fire protection, also the small dimensions of the optical fiber (cladding diameter is only 250 µm), at the same time, with a potentially long length (up to tens of kilometers), make it possible to create arrays of sensors in one fiber and its embedding in various materials including composite materials for various purposes. At present, many tasks can be solved in science and technics with the help of FOSs. They are useful for measuring temperature [26,27,28], deformation [29,30], rotation speed [31], acceleration [32,33], vibration [34,35], liquid level [36,37], and other parameters [38,39,40,41,42,43,44,45]. Separately, it is worth highlighting the ability of optical fiber to register acoustic vibrations in various environments [46,47,48], as well as vibrations of various objects and mechanisms [49,50,51], which forms the basis of acoustic diagnostics of technical objects, in particular, power plants, where non-invasive monitoring of the state of individual units is required, including GTUs used in GT engines, liquid rocket engines, and gas compressor stations.

The aim of the work is to substantiate the possibility of non-invasive acoustic monitoring and study of GTU using fiber-optic sensors based on the Mach–Zehnder interferometer (MZI) to determine the turbine’s rotor speed and determine the presence of defects in its parts.

## 2. Theory

A spectral component’s analysis of the generated by the GTU signals shows that two ranges can be distinguished in them. The first is located in the range from 10 to 1000 Hz and allows to register of signals that occur in the case of the normal operation of the system. For example, shaft speed or rotor resonant frequencies. The frequency range should be expanded to a higher frequency region in the case of some specific defects, for example, various geometry violations of the turbine’s and compressor’s flow path, local violations of the flow velocity field around the rotor blades due to changes in geometry, etc. [17]. Higher-order harmonics and beats can be registered in it, arising from the deviation of elements from the standard operating mode. For GTU, one of the manifestations will be an increase in the sound component generated by the rotation of the turbine disk with the number of blades *N*. Its frequency is determined as the product of the intrinsic rotor speed *f_turb_* and the number of turbine blades [52]:

(1) $$f=f_{turb}\cdot N.$$

One of the main factors limiting the service life of GTU is the destruction of turbines and pumps due to the destruction of the unit’s rolling bearings due to abnormal vibrations. They were caused, among other things, by the mechanical deterioration of its individual parts. For example, bearings in the middle and on the blades, as well as the blades themselves, the rotation frequency of which can reach tens of thousands of Hertz.

A microphone can be used as an acoustic signal recorder. However, this sensor is not suitable for use in all cases. It requires a power supply, is sensitive to electromagnetic interference and humidity, and has a large size, which requires additional space for its installation. In comparison, fiber sensors are very compact and do not have these drawbacks. For the described task of recording audio signals, several types of fiber sensors can be used.

**Figure 1:**
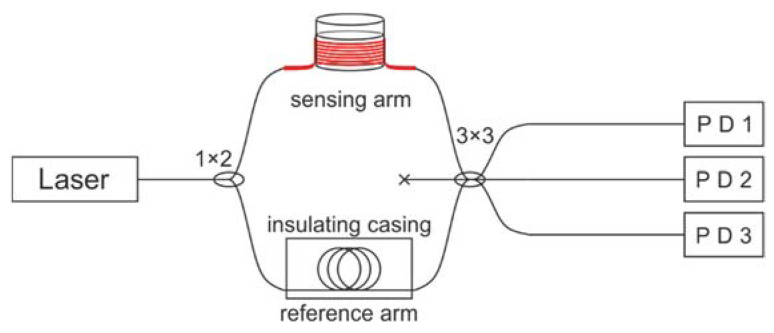
Scheme of sensor based on the MZI.

For the timely diagnosis of defects in the most loaded elements of a GTU, it was proposed to use MZIs fixed directly at the locations of bearings and blades. The frequency range of the MZI sensor is limited by the photoreceiver and the ADC. In our experiment 1 MHz range was possible. The scheme of the used fiber-optic vibration detector is shown in Figure 1.

The emission from a narrow-band laser was divided in the splitter in equal proportion. The first part was sent to an isolated reference arm. The second went to the sensing arm. This arm was installed on the monitored element and reacted to its deformation by phase shift caused by the fiber length deviations. After the radiation from both arms interferes in a 3 × 3 splitter. Two outputs with the photodetector 1 (PD1) and the photodetector 2 (PD2) were collected by ADC. They have a 2*π*/3 phase shift between each other. The unwrapped incoming signal is then calculated according to the formulas [53,54]:

(2) [IPD1(t)=I1+I2+2I1·I2·cos2(πλΔ(t)+φ0),    IPD2(t)=I1+I2+2I1·I2·cos2(πλΔ(t)+φ0+2π3).

(3) $$\Delta \varphi (t)=\int_{0}^{t}\left[S_{1}\left(t\right)\cdot {S_{2}}^{\prime }\left(t\right)-S_{2}\left(t\right)\cdot {S_{1}}^{\prime }\left(t\right)\right]dt$$

where $S_{1}\left(t\right)=I_{PD1}\left(t\right)-I_{PD2}\left(t\right)$, $S_{2}\left(t\right)=I_{PD1}\left(t\right)+I_{PD2}\left(t\right)$.

The frequency range of the recorded signals corresponds to half of the sampling frequency of the ADC. The obtained signal phase values at each time Δφ(*t*) can be analyzed by various digital signal processing methods, for example, it is possible to draw spectrograms of the received signal. In the absence of mechanical wear, the main recorded frequencies will be the rotational speeds and their harmonics.

## 3. Setup

**Figure 2:**
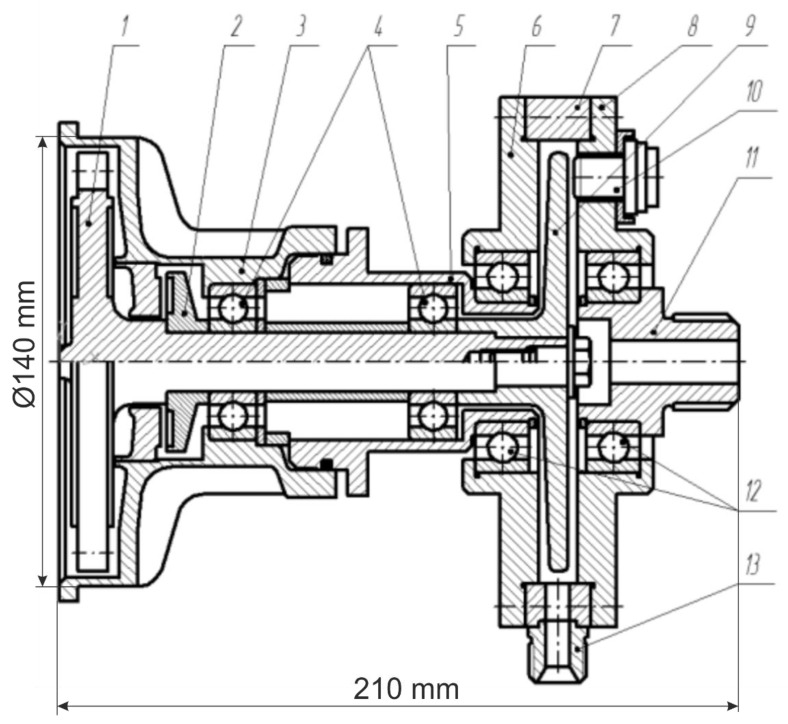
General view of the GTU working section. 1—turbine rotor; 2—impeller water seal; 3, 5—parts of the turbine housing; 4—turbine bearings; 6, 7, 8—parts of the hydraulic brake housing; 9—hydraulic brake disc; 10—speed sensor; 11—hydraulic brake frame; 12—bearings of the hydraulic brake housing; 13—fitting for connecting the line for supplying working fluid to the hydraulic brake.

**Figure 3:**
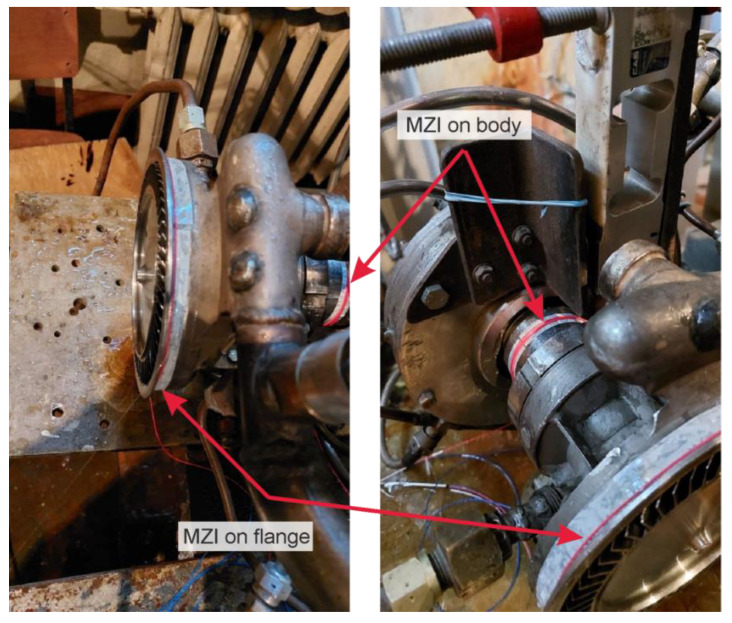
Location of fiber optic sensors on the GTU.

The object of the study was a GTU, consisting of an active supersonic partial turbine and a disc hydraulic brake used as a load and a torque meter. A general view of the GT with sensors is shown in Figure 2 and Figure 3.

On a GTU of this type, at a pressure level before the turbine of up to 10 MPa, a maximum rotational speed level of up to 50 kHz is achievable. This model GTU is used for educational purposes and is operated at frequencies not exceeding 500 Hz in order to increase the service life. Air was used as the working medium during the experiments. The main parameters of the GTU are shown in Table 1.

**Figure 4:**
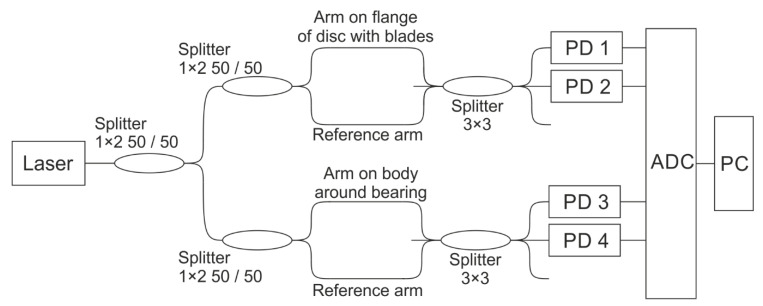
Scheme of the setup.

Fiber-optic sensors were installed on the flange near the disc with blades and on the housing around the rolling bearing. The experiment was carried out twice. Firstly, with the use of new bearings. Secondly, with bearings after service life. The scheme of the experiment is shown in Figure 4.

The experiment was carried out with the following parameters:

Turbine rotor speed up to 500 Hz.

During the experiment, the signal was recorded for each measurement (mode of operation) with a recording time of 10 s with ADC sampling of 500 kHz.

The emitter source was a narrow-band laser NKT Photonics Koheras BASIK with linewidth < 0.1 kHz.

The length of the fiber section on the bearing and blades: 80 cm ± 5 cm.

The calibration of the setup consisted in verifying the part of power quantity going through each of the MZI’s arms, as well as the registered power value by each of the photodetectors. For the best contrast, the power in both arms should be approximately the same; also for these purposes, the power entering the photodetectors must be the same. The verification is performed by pairwise enabling/disabling the MZI’s arms.

The power control on the photodetector was verified by hit impact, so that the interference is covered completely by the range of the ADC and the photodetector and that it is stable in time for subsequent normalization to [−1…1] and phase unwrapping.

## 4. Analysis

As mentioned in the previous section, the experiment was carried out twice: using new bearings, and also with bearings after the service life. In both cases, data were obtained for the following operating modes: before the start of engine operation (external noise and vibrations), engine acceleration, nominal operation at certain frequencies, and turbine rotor shutdown.

**Figure 5:**
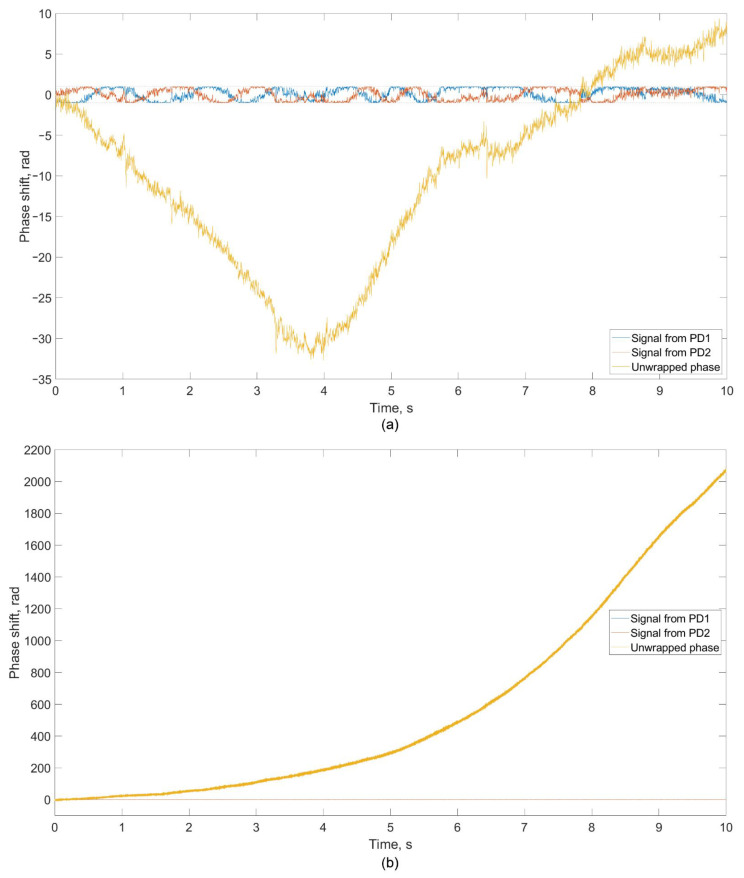
Received data from photodetectors PD 1 and PD 2, as well as the reconstructed phase of the registered emission: (**a**) before starting the engine, (**b**) for the acceleration mode.

**Figure 6:**
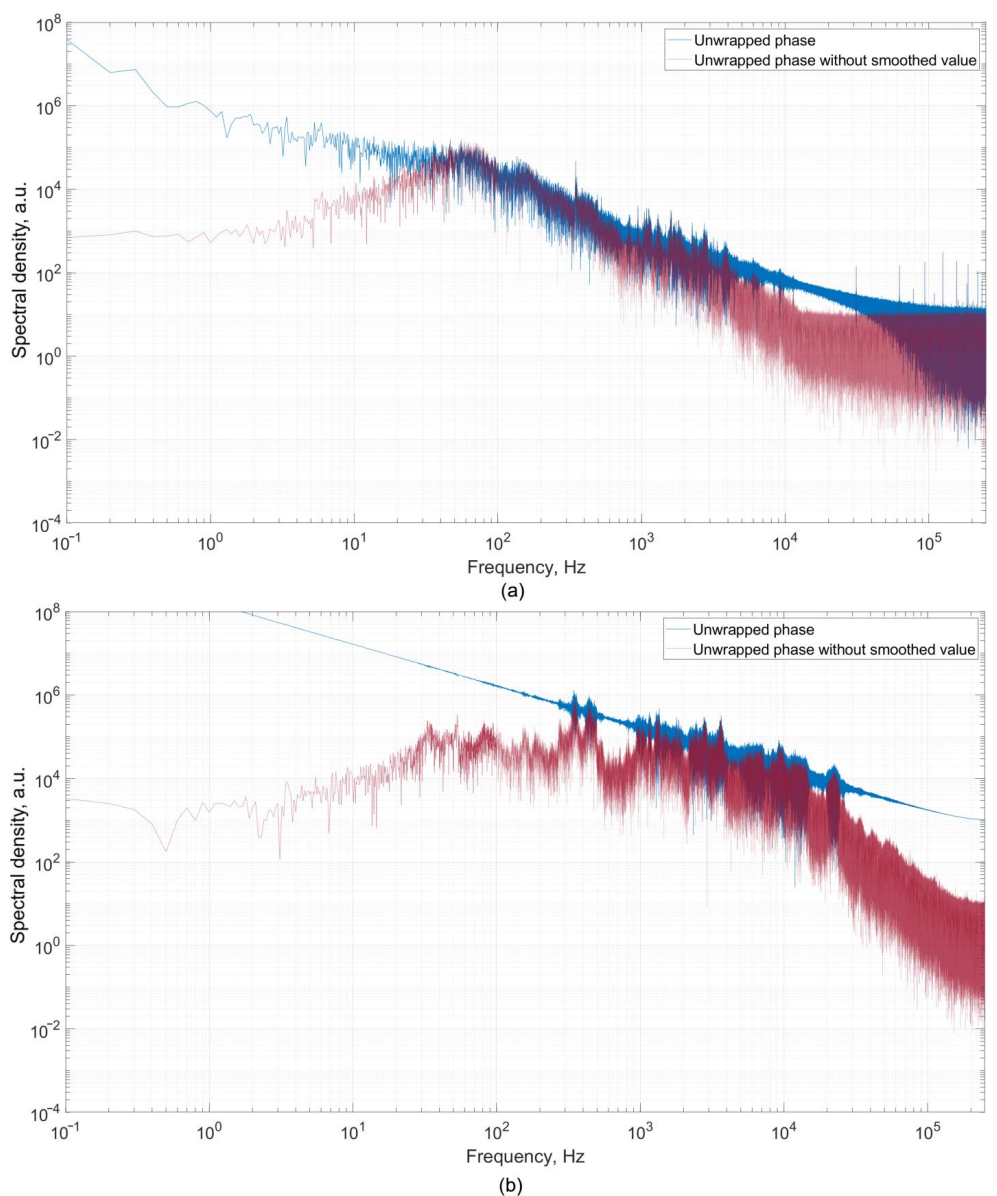
Signal spectrum before starting the engine (**a**) and at the stage of engine acceleration (setting the mode) (**b**). Blue—unwrapped phase, red—moving average for 2 s was subtracted.

The measurement before starting the engine was carried out to obtain the noise level in order to be able to further deduct it from informative data. An example of the received data from photodetectors PD 1 and PD 2 is shown in Figure 5a. The presented signal form is typical for all measurements. The data recorded by the photodetectors were normalized in the range [−1; +1] for the possibility of applying the algorithm according to Formula (2) in order to unwrap the phase. When the GTU is switched off, the change in the difference between MZI’s arms, that is, the phase shift, occurs without a dedicated general direction of signal phase change. The shift has a random direction and over time can return to zero. The resulting signal’s spectrum in the absence of rotor operation does not have pronounced peaks at specific frequencies (Figure 6a). Spectral analysis was carried out for the unwrapped signal according to Formula (3). Further in the article, the graphs and processing results for each of the experiments are presented for the restored signal.

At the stage of starting the engine (setting the mode), the photodetectors registered a strong monotonic increase in the phase shift, which is shown in Figure 5b. Analysis of the spectrum of the recorded signal showed the presence of peaks at different frequencies (Figure 6b). The general dynamics of an increase in the signal level and noise level with the start of engine operation are traced.

**Figure 7:**
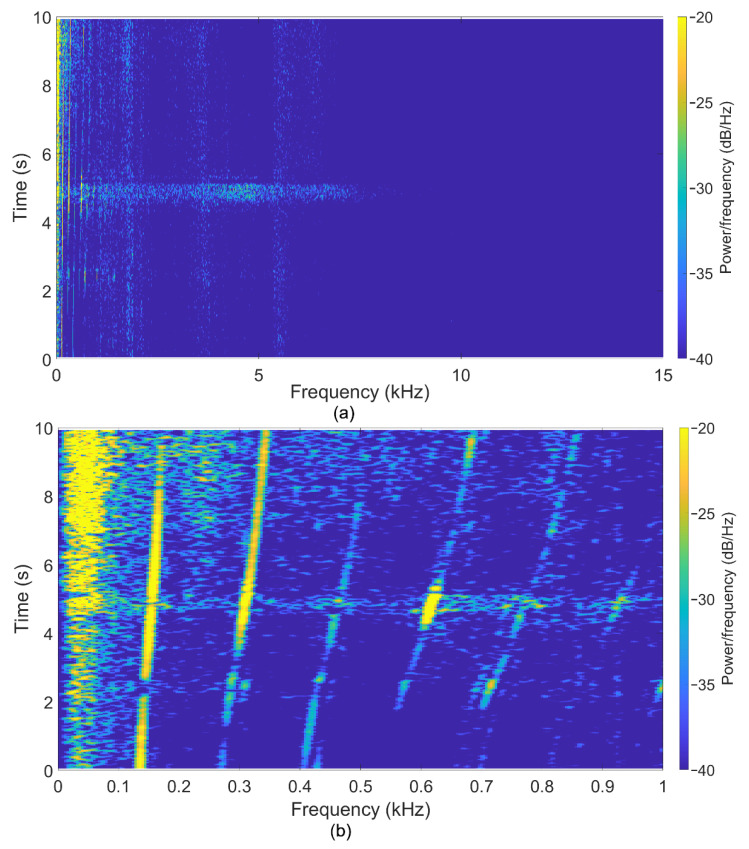
Spectrogram of the signal for the regime with a rotor speed frequency range from 130 to 175 Hz during experiments with new bearings and blades. (**a**) spectrograms up to 15 kHz, (**b**) low-frequency range with the fundamental frequency and its harmonics.

New previously unused blades and bearings were used during the experiments to determine the normal, nominal indicators during acoustic monitoring. The turbine rotor speed and its harmonics are visible in the signal spectrogram in Figure 7. Also, there are no high-frequency noises indicating blade defects. The peak’s shift corresponds to a change in the turbine rotor rotational speed, which also confirms the possibility of using this type of sensor for the non-invasive acoustic control of GTU.

The plots from two MZIs: on the flange (photodetectors PD1 and PD2) and on the housing at the bearing location (photodetectors PD3 and PD4) show similar results for the recorded frequencies, with the exception of a slight difference in the amplitude of the incoming signal. In addition to previously unused bearings, experiments were carried out with elements after their service life.

**Figure 8:**
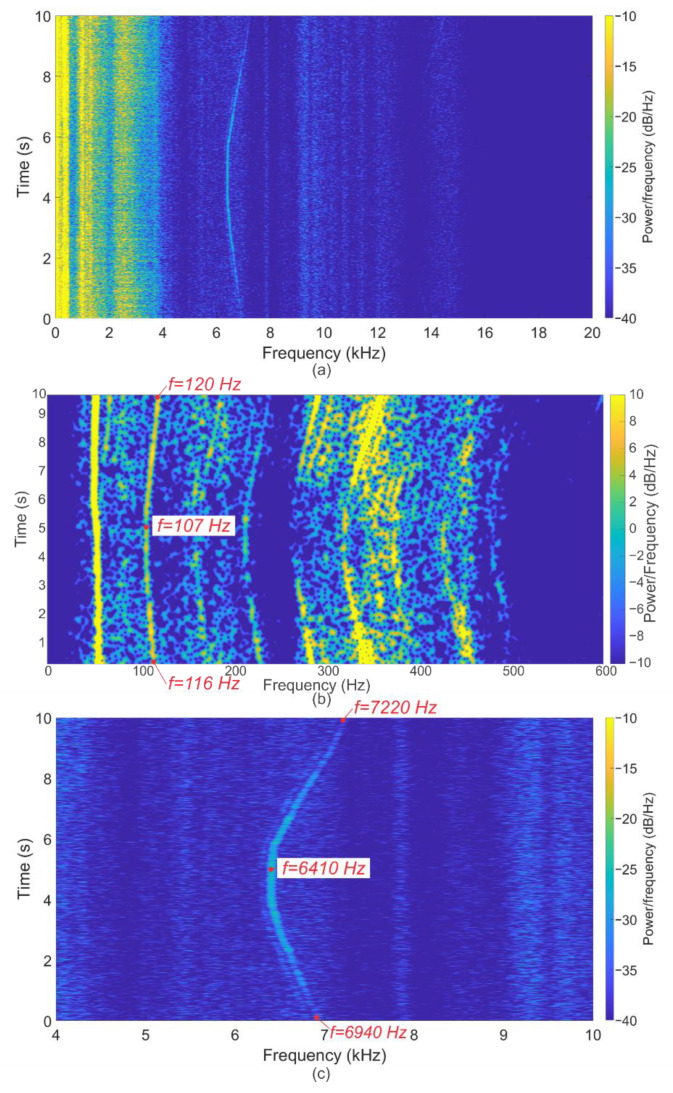
Spectrogram of the signal for the regime with a rotor speed with frequency in a range from 107 to 120 Hz during experiments with bearings and blades after their service life. Spectrogram for the entire measurement time for the frequency range up to 30 kHz are shown (**a**), the low-frequency region with the fundamental frequency in a range from 107 to 120 Hz and its harmonics, as well as interference from the 50 Hz power supply (**b**), as well as the peak from blade noise (**c**), the frequency of which is greater than N times (N = 60, the number of turbine blades).

One of the operating modes was a mode with frequency in a range from 107 to 120 Hz. Analysis of the spectrograms shows peaks in the region of 50 Hz, caused by interference from the power supply almost throughout the entire regime. A group of peaks from harmonics of the central frequency is also distinguished. The most interesting result is the peaks in the region of 6.7 kHz. These values are in good agreement with Formula (1), which confirms the presence of acoustic noise from the blades, indicating their condition with wear and possible defects. The resulting spectrograms are shown in Figure 8.

**Figure 9:**
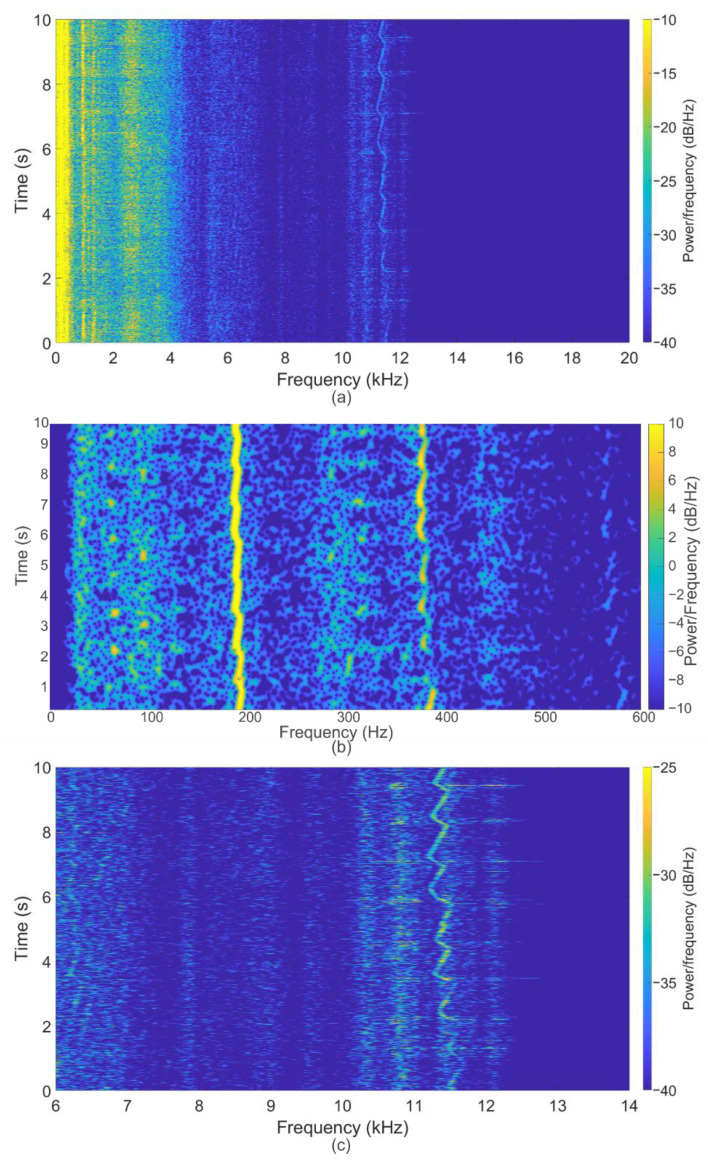
Spectrogram of the signal for the regime with a rotor speed frequency ~192 Hz during experiments with bearings and blades after their service life. Spectrograms for the entire measurement time for the frequency range up to 30 kHz are shown (**a**), the low-frequency region with the fundamental frequency ~192 Hz and its harmonics (**b**), as well as the peak from blade noise (**c**), the frequency of which is greater than *N* times (*N* = 60, the number of turbine blades).

The next mode of operation had a center frequency of ~192 Hz. The obtained spectrogram is shown in Figure 9. The spectrogram clearly shows a peak at ~192 Hz (coinciding with the turbine operating mode) throughout the entire mode, as well as its harmonics at 384 Hz and higher at separate time intervals during recording with a reduced intensity amplitude. When expanding the range of analyzed frequencies, a sawtooth signal appears at 11.5 kHz throughout the entire mode, which also corresponds to Formula (1).

It can be seen that after a long service life, defects may appear, caused by, among other things, blades and other elements, which leads to the appearance of additional high-frequency noise corresponding to Formula (1).

## 5. Conclusions

A method using fiber-optic Mach–Zehnder interferometers as sensors for non-invasive acoustic diagnostics of a model GTU is presented. It allows controlling the turbine rotation frequency at any time in various operating modes. Phase unwrapping allows setting the real phase shift of the recorded signal because without unwrapping, it lies in a range [−1…+1]. The spectral analysis method allows registering frequencies up to 0.5 ADC sampling. In our case, we used an ADC with a frequency sampling rate of 500 kHz, which is not the limit, but sufficient to record the rotor speeds of the experimental axial turbine. The method showed that the spectrogram of the recorded signal on the new adjusted GTU contains frequency components corresponding to the main rotor speed, as well as its harmonics, which gradually decrease in amplitude. When testing the installation after a long operation period, high-frequency noise components begin to appear on the spectrogram, corresponding to an increased main rotor speed by N times, where N is the number of turbine blades, the amplitude of which significantly exceeds the level of other harmonics. This allows monitoring of the installation status and determination of the moments for the replacement of the bearings and the GTU tuning.

A further direction of research will be developing a GTU automatic control system with feedback from the recorded parameters to the turbine rotor speed control unit and works devoted to the development of a compact portable design of fiber optic primary recorders, intermediate converters, and recorders.

## Figures and Tables

**Table 1:** Parameters of the experimental GTU.

Parameter, Designation	Value
Number of nozzles, *n*	2
Estimated expansion ratio, *δ*	10
Nozzle angle, *α_c_*, grad	20
Nozzle velocity coefficient when operating on a single-phase working medium, *ϕ*	0.9
Degree of partiality, *ε*	0.04
Average diameter, *D_p_*, mm	125
Impeller width, *b*, mm	12
Blade height, *h*, mm	14
Blade input angle, *β_b1_*, grad	27
Blade outlet angle, *β_b2_*, grad	27
Estimated speed coefficient of the nozzle array when operating on a single-phase working medium, *ψ_p_*	0.823
Turbine wheel diameter, *D_turb_*, mm	140
Number of blades, *N*	60

## Data Availability

The data presented in this study are available on request from the corresponding author.
